# MoleGear: A Java-Based Platform for Evolutionary De Novo Molecular Design

**DOI:** 10.3390/molecules24071444

**Published:** 2019-04-11

**Authors:** Yunhan Chu, Xuezhong He

**Affiliations:** Department of Chemical Engineering, Norwegian University of Science and Technology, N-7491 Trondheim, Norway; yunhan.chu@ntnu.no

**Keywords:** de novo design, evolutionary algorithm, drug molecules, fitness, multi-objective function

## Abstract

A Java-based platform, MoleGear, is developed for *de novo* molecular design based on the chemistry development kit (CDK) and other Java packages. MoleGear uses evolutionary algorithm (EA) to explore chemical space, and a suite of fragment-based operators of growing, crossover, and mutation for assembling novel molecules that can be scored by prediction of binding free energy or a weighted-sum multi-objective fitness function. The EA can be conducted in parallel over multiple nodes to support large-scale molecular optimizations. Some complementary utilities such as fragment library design, chemical space analysis, and graphical user interface are also integrated into MoleGear. The candidate molecules as inhibitors for the human immunodeficiency virus 1 (HIV-1) protease were designed by MoleGear, which validates the potential capability for *de novo* molecular design.

## 1. Introduction

Computational chemistry plays an important role in the design of new drug-like molecules [1,2,3,4,5], catalysts [6,7,8], and novel solvents of ionic liquids [9,10,11]. De novo molecular design has been an active research area of drug design/discovery over the last decades, and many approaches such as LUDI [12], LEA3D [13], Flux [14,15], and pharmacophore-linked fragment virtual screening (PFVS) [16] have been developed by using protein and ligand structures. The ligand-based approaches have wider applicability, especially when the three-dimensional (3D) structure of the target is not available [17]. It should be noted that the chemical space is huge, which makes it difficult to search the appropriate structure through the whole space. Thus, strong effort has been put on the development of effective heuristic algorithms for the searching and optimization purposes. Evolutionary algorithms (EA) mimic natural evolution’s ability to produce functional objects (e.g., structures, parameters, and programs) with the use of analogous mechanisms—reproduction, mutation, recombination (crossover), and selection. By applying EA to the molecular design fields, a diverse chemical space can be searched to provide optimal, or near-optimal solutions to a wide range of objectives. So far, many studies have reported the use of EA tools for computer-aided de novo molecular design [6,15,17,18,19,20,21,22]. Evolutionary algorithms use fitness functions to determine the surviving structures, which will be used in the next generation population. Different fitness functions are reported in the de novo design tools, for example, the similarity-driven fragment-based evolutionary approach was reported by Kawai et al. [17], and Flux used a similarity index [15]. The docking scoring method based on AutoDock [23] and AutoDock Vina [24] (hereafter refers to Vina) provides an efficient way to calculate the fitness of structure-based molecules. These approaches can generate suitable structures. However, a combination of different fitness functions can potentially provide a higher flexibility for de novo design of drug-like molecules. In this work, a multi-objective fitness function including both docking score and similarity score is introduced to the evolutionary de novo design. A unique Java-based platform, MoleGear is developed based on a Java script using different tools such as Balloon [25], Open Babel [26] and MolConverter/Cxcalc (ChemAxon), and AutoDock and Vina. In order to evaluate the functionality of MoleGear, the design of candidate molecules of the human immunodeficiency virus 1 (HIV-1) protease inhibitors was conducted. Different fitness functions based on the receptor-based and ligand-based scoring strategy as well as the combination were used to score the newly designed molecules. Finally, the best candidate molecule with a structure and conformation similar to indinavir was discovered.

## 2. Results and Discussion

### 2.1. Dataset

The compounds in the National Cancer Institute (NCI) diversity set [27] has been widely used in docking-based virtual screening studies [28,29,30], and specifically as a benchmark dataset for comparing AutoDock and Vina in the application of screening for inhibitors that are active against human immunodeficiency virus (HIV-1) protease [31,32].

**Figure 1:**
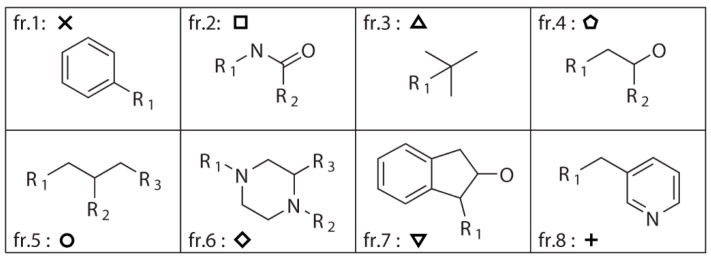
Fragments split from the structure of indinavir using the fragment library tool of MoleGear leading to 4 side chains (fr.1, fr.3, fr.7, and fr.8) that contain single R-group, and the scaffolds of the fr.2 and fr.4 have two R-groups, while fr.5 and fr.6 have three R-groups.

A dataset consisting of 1990 compounds selected from around 140,000 compounds in the National Cancer Institute diversity set is used for drug design, which covers a broad range of chemotypes. The application of fragment library tools on the dataset in MoleGear generates a library consisting of 1151 fragments including 599 side chains with one R-group and 552 scaffolds. Among those scaffolds, 462 have two R-groups, 75 have three R-groups, 14 have four R-groups, and 1 has five R-groups (see the representative graphs graphical presentation in Figure 1). To build up the dataset, only the compounds containing the atoms of C, N, O, S, P, F, Cl, Br, and I were selected. The fragments that were charged or possessed more than 16 atoms or three fused rings or at least one ring with more than 7 atoms were excluded according to self-defined rules. Moreover, the fragments that possessed atoms other than C, N, O, and S were also excluded. The same fragmentation routine was applied to the structure of indinavir, and 8 fragments (fr.1–fr.8) including 4 scaffolds and 4 side chains are obtained as shown in Figure 1. It was found that 284 components in the NCI diversity set cover the fragment of fr.1, and at least more than 7 components cover the fragments of fr.2–fr.5. In total, 98 fragments including those fragments appeared >7 times in the NCI dataset together with the fragments of fr.6–fr.8 in Figure 1 were selected for drug design in this work.

### 2.2. Case Study—MoleGear for Drug Design

**Figure 2:**
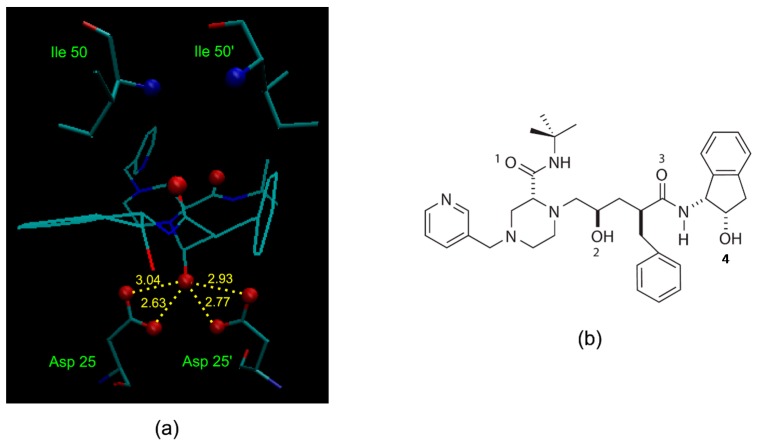
(**a**) Active site of HIV-1 protease in complex with its inhibitor indinavir (Protein Data Bank (PDB) structure 1HSG). Specific interactions between the enzyme and the inhibitor include the hydroxyl group (O2 in (**b**)) hydrogen bonding to the carboxyl groups of the essential Asp 25/25’ enzymic residues (hydrogen bonding distances are shown in angstroms), and the amide oxygens (O1 and O3 in (**b**)) of the inhibitor hydrogen bonding to the backbone amide nitrogen of Ile 50/50’ via a mediating water molecule. (**b**) Structure of indinavir with the numbering of oxygen atoms.

A simple illustration of HIV-1 protease active site in complex with an indinavir—one of its potent and orally bioavailable inhibitors—at a resolution of 2.0 Å (Protein Data Bank (PDB) structure 1HSG [33]), is shown in Figure 2a. One important interaction between the enzyme and the inhibitor is the critical hydroxyl group (refers to O2 in Figure 2b) that forms a hydrogen bond to the carboxyl groups of the catalytically active aspartic acid. Incorporation of structural isosteres as replacements of the hydroxyl group may lead to compounds that are potent and selective to HIV-1 protease.

**Figure 3:**
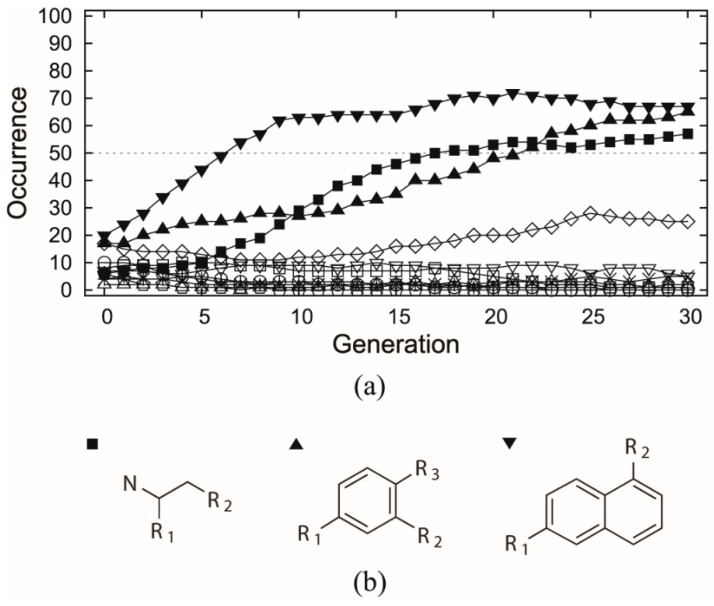
(**a**) Occurrence versus generation averaged by 6 receptor-based evolutionary algorithm (EA) experiments, which involves the 8 indinavir related fragments (unfilled shapes) as well as the fragments that were ever selected over 50 times (filled shapes). The samples correspond to the fragments marked of same shape in Figure 1 and (**b**) fragments that were selected over 50 times by at least one generation, including two scaffolds associated with two R-groups and one scaffold associated with three R-groups.

**Figure 4:**
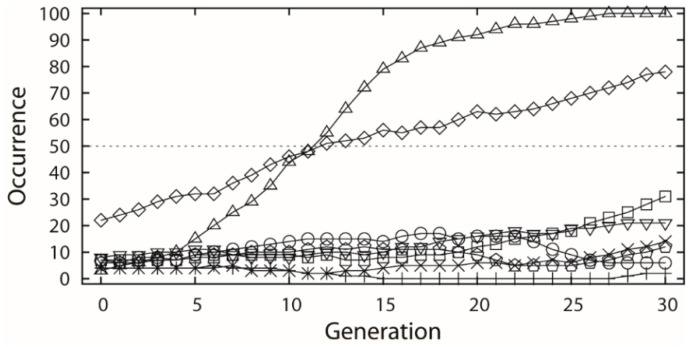
Occurrence along generation averaged by the 6 ligand-based EA experiments, which involves the 8 indinavir related fragments. The samples correspond to the fragments marked of same shape in Figure 1.

The selected dataset was applied to both receptor- and ligand-based evolutionary de novo design of the novel inhibitors for HIV-1 protease. For the receptor-based design, the 1HSG HIV-1 protease structure obtained from the Protein Data Bank (PDB) [33] was used as the receptor, and the binding free energy predicted by docking based on AutoDock 4.2 was used to estimate the fitness of the candidate structures. For the ligand-based design, the similarity to the indinavir structure based on the 82 descriptors covering seven 3D and eight 2D categories (see Table 1) was used as fitness function. Both the receptor- and ligand-based designs used a population size of 100 individuals and maximum 30 generations, and each type of design was repeated 6 times using different random numbers. Ideally, the indinavir structure should be found by the EA runs as all the related fragments are included. However, this is almost impossible in practice due to a huge combinatorial space. Thus, we only inspect the cases in which all relevant fragments occur in the generations. The occurrences of each indinavir related fragment were averaged among the 6 experiments along with the generations, as shown in Figure 3a and Figure 4, respectively.

In the receptor-based design, all the 8 indinavir related fragments (referring to Figure 1) were selected by the EA initial generation (Figure 3a), but none of them comes out on the top in the end despite the relatively high persistence of fr.6. In contrast, significant selection goes to the three fragments derived from the NCI diversity set (referring to Figure 3b) which in average have been used over 50 times in the final generation of the evolution runs. This indicates that building blocks derived from active ligand molecules (here refers to indinavir) can potentially lead to good but not necessarily the best candidate molecules.

In the ligand-based design, all the 8 indinavir related fragments (referring to Figure 1) were also chosen by the first generation of EA (Figure 4). However, the fragments of the fr.3 and fr.6 were mostly used by next generations of the EA runs, which may be due to the nature of the similarity pressure. Besides the evident increase of the occurrences of the fr.3 and fr.6, other fragments also got a moderate or slight increase in growth except the fragments fr.5 and fr.8.

**Figure 5:**
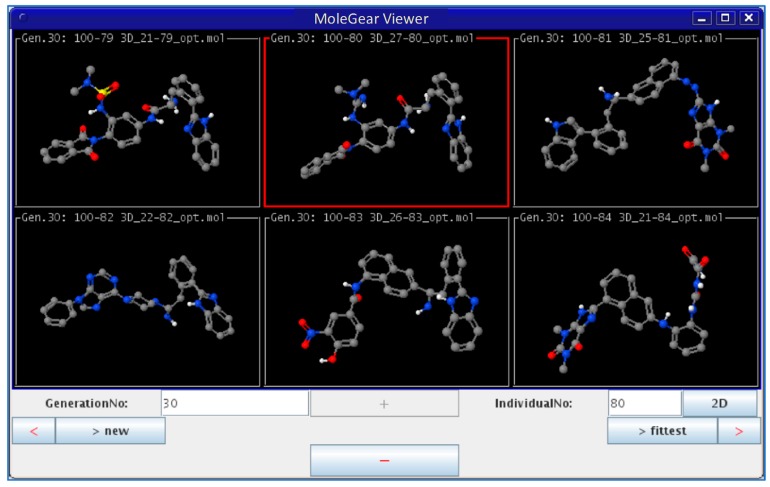
Graphical view of outcome from an EA run by MoleGear.

**Figure 6:**
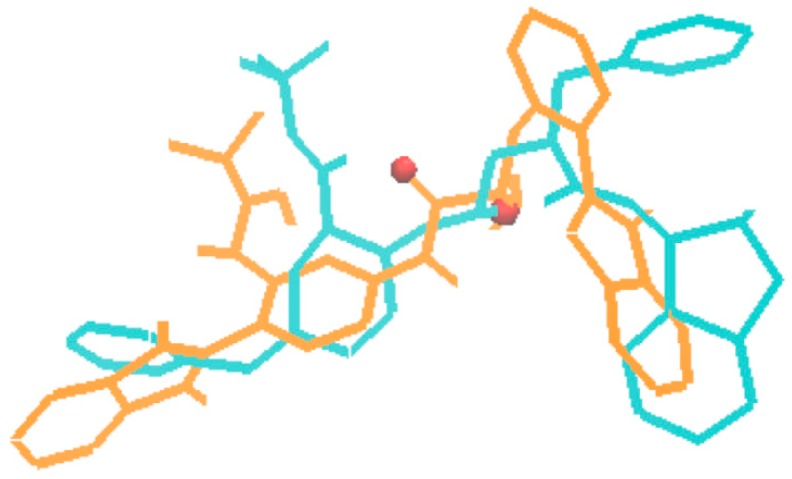
Superposition of the indinavir structure (in cyan) cut from the 1HSG complex with the AutoDock 4.2 predicted binding mode of the molecule no.80 (in orange, marked with red frame in Figure 5).

**Figure 7:**
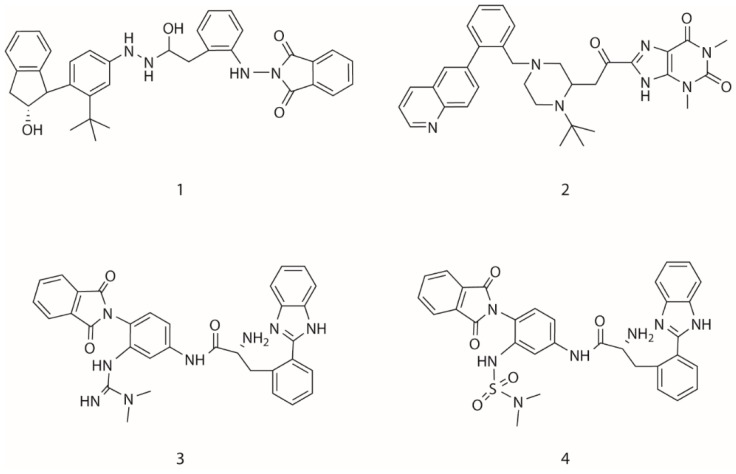
The candidate ligands for HIV-1 protease designed by MoleGear from the EA runs using the multi-objective function combining half-to-half the receptor- and ligand-based scoring strategy.

In the third type of design, a combination of the receptor-based and ligand-based scoring strategies was conducted to define a half-to-half weighted multi-objective function. A graphical view on the outcome of one representative evolution experiment is shown in Figure 5. The molecule of no.80 (marked red frame) in the population appeared in the last generation, which is considered as the highest fitness of both the binding affinity to HIV-1 protease and the similarity to indinavir. A superposition between the indinavir inhibitor cut from the 1HSG complex and the AutoDock 4.2 predicted binding mode of the no.80 molecule is shown in Figure 6 (i.e., the molecule 4 in Figure 7). Overall, this molecule simulates indinavir well both in structure and conformation, and a carbonyl oxygen is attempting to perform similar function of the hydroxyl oxygen of indinavir although there are still some differences in the conformation. Some other ligands for HIV-1 protease designed by MoleGear from the relevant experiments are shown in Figure 7, which are also considered as good candidates. It should be noted that the developed MoleGear provides a general capability of de novo molecular design, and the integration with other methods such as machine learning and artificial intelligence can be further conducted in the future work to enhance the design power. Moreover, testing the capability of the developed platform for designing of intrinsically disordered protein–ligand complex or exploring inactive compounds [41] can also be performed.

## 3. Methods

**Figure 8:**
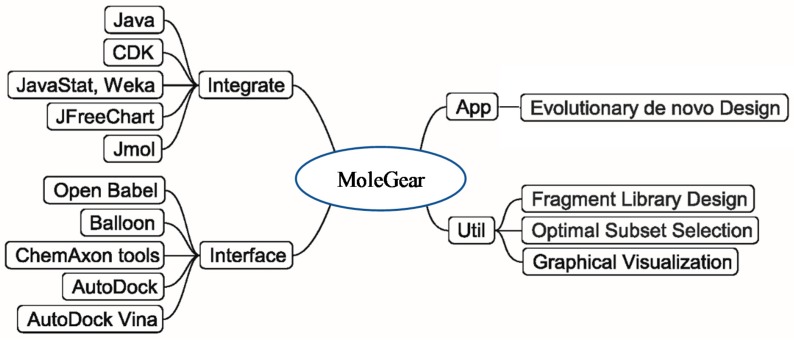
General structure of MoleGear.

The general structure of MoleGear is shown in Figure 8, and evolutionary de novo design represents a major function that provides a stochastic way for exploration of chemical space. A suite of fragment-based operators such as crossover, mutation, and growing are used in MoleGear for the assembly of new molecular structures based on a graph-based molecular representation provided in the chemistry development kit (CDK) [42].

The exploitation of chemical structure space by MoleGear can be guided by fitness functions in accordance with different strategies—the affinity of molecules binding to a protein target, the similarity of novel molecules to an available active molecule, or quantitative structure–activity/property relationship (QSAR/QSPR). These scoring strategies are combined as an appropriate multi-objective fitness function for the estimation of the integral quality of novel molecules. Before starting any fitness function computation, an acceptable initial 3D structure of a molecule is required. MoleGear is interfaced to the programs of Balloon [25], Open Babel [26], and MolConverter/Cxcalc (ChemAxon) to provide different methods for 3D conformational search. It should be noted that MoleGear only provides the interfaces to the external software.

AutoDock and Vina can be called by MoleGear to provide a receptor-based scoring based on binding free energy estimation. Moreover, a set of molecular descriptors can be obtained from CDK package that is integrated into MoleGear. Therefore, ligand-based scoring functions based on molecular similarity or QSAR can be defined. Currently, the partial least square regression (PLSR) model in the Weka package is implemented in MoleGear for QSAR analysis.

Besides the major function of the evolutionary de novo design, other complementary utilities such as fragment library design and chemical analysis of a molecule set (i.e., Optimal Subset Selection) are also supported by MoleGear as shown in Figure 8. Moreover, MoleGear provides graphical user interface (GUI) to visualize molecular population and their property space. Some of these functions are implemented by integrating Java packages of Jmol and JFreeChart (for chemical structure and space visualization, respectively) as well as Weka and JavaStat for statistical analysis [6].

### 3.1. Evolutionary Algorithm for De Novo Design

**Figure 9:**
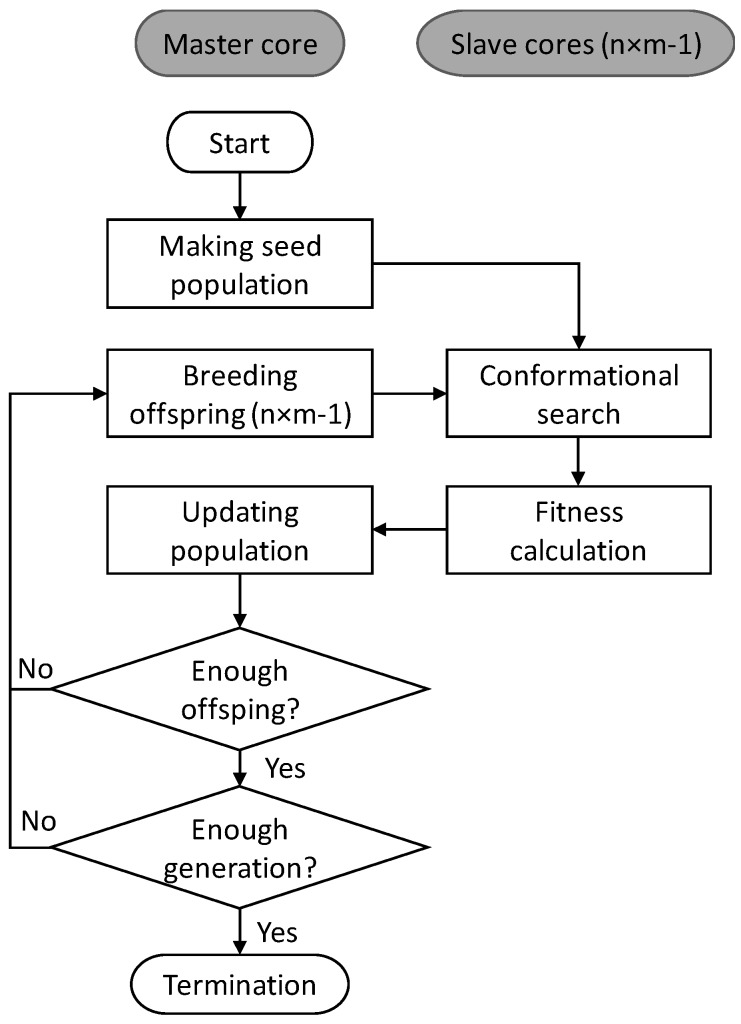
Evolutionary scheme of MoleGear.

The basic scheme of EA in MoleGear is illustrated in Figure 9. A seed population including *k* molecules is initially constructed either from an available set of chemical structures [18] or a fragment library. All the structures are by default saturated with hydrogens and subjected to a conformational search performed by 3D builders. The structure with the lowest energy among a pre-defined number of searched conformers of each molecule is saved and scored by a fitness function. After that, the optimization cycle consisting of four main steps starts: (i) new offspring molecules are bred by structural operators such as growing, crossover, and mutation; (ii) conformational search and fitness calculation are conducted for the generated offspring molecules; (iii) the population is updated by replacing the least competitive structures with more fit offspring molecules. The optimization cycle continues until a pre-defined number of offspring structures have been produced, and a new generation is obtained by the combination of the offspring and the current population. The population evolves over generations until a termination criterion is satisfied (e.g., maximum of generations or a minimum number of satisfying solutions) or exhaustion sets in (e.g., no improved solution is found within a limited number of successive searches).

It should be noted that MoleGear supports parallel implementation of the conformational search and fitness calculation of the EA either on a cluster-type architecture with the Message Passing Interface (MPI) or a general multicore machine through multithreading based on the OpenMPI environment. The parallelization scheme is implemented by a cluster with multiple nodes. The *n* nodes are allocated to implement the overall computation job, and each node contributes m processor cores. The Core one on the master node generates (n × m − 1) molecular structures and transfers them simultaneously to the (n × m − 1) slave cores. While the slave cores are computing the fitness of the current batch of molecules, the Core one prepares the next batch of new (n × m — 1) molecules and then receives the previous batch of calculated molecules to update the population. In case of expensive and large-scale molecular computations, substantial time can be saved with parallel implementation compared to serial implementation.

### 3.2. Molecular Assembly

*Molecular representation*: A graph-based molecular representation method provided from CDK is employed in MoleGear. One molecular diagram is parsed to a set of atom and bond objects with connectivity information stored in a data structure container. The graph-based representation has a good resemblance of the constitution of a chemical structure, which can easily be manipulated by human knowledge. It should be noted that the properties of a chemical structure are highly dependent on the 3D structure, and thus an appropriate 3D structure is usually required which is generated by an explicit program like Balloon in MoleGear.

*Building blocks*: Both atoms and fragments can be used as basic building blocks for the assembly of candidate structures. Atom-based approaches are superior to fragment-based methods in the generation of a diverse structure space, but not good at generating a chemically sensible space. Fragment-based strategies create chemically sensible structures by using fragments that are commonly occurring in available drug molecules, which can significantly reduce the search space. In MoleGear, the definition of fragment is not that rigid, and can vary from an atom to a polycyclic ring system. The fragments can be pre-managed with a fragment library so that each building block selected by the EA can be well tracked.

**Figure 10:**
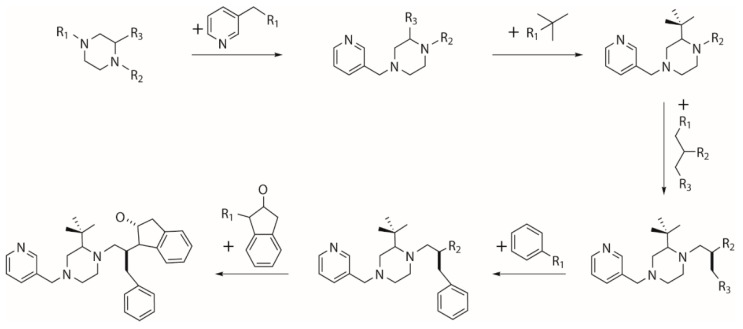
Generation of seed population by growing from an initial “core” scaffold.

**Figure 11:**
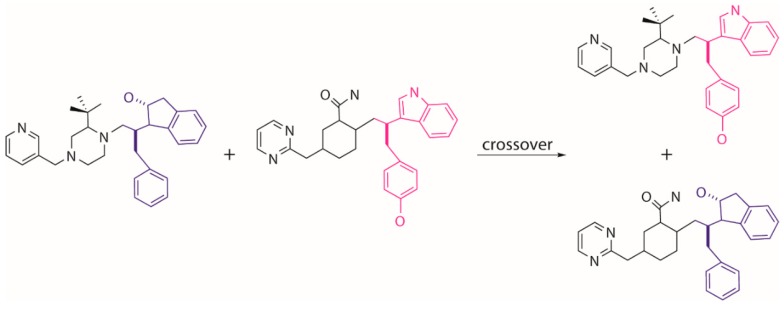
Generation of novel molecules by crossover of two parent structures.

**Figure 12:**
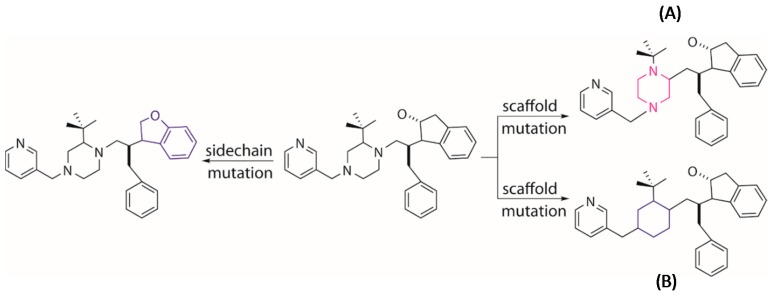
Generation of novel molecules by mutation of a parent structure through changing the position of a local scaffold (**A**) or replacing a local scaffold (**B**) or replacing side chain (**left**) with a new entry.

*Structural operators*: The choice of EA for chemical space exploitation implicitly makes genetic operators to be responsible for the manipulation of molecular structures. With the graph-based molecular representation described above, three operators (i.e., growing, crossover, and mutation) are implemented in MoleGear to assemble novel candidate molecules, as illustrated in Figure 10, Figure 11 and Figure 12.

The growing operator builds a new molecule from an initial “core” scaffold which is randomly picked from a coupled fragment library and contains at least two substitution points (Figure 10). The molecular fragments are added to the empty substitution points of the “core” scaffold and previously added moieties until no empty substitution point is left. To prevent growing too large molecules, all added fragments are ordered to process substitution points no more than the moieties they attach to. A seed population of novel compounds is thereby generated by repeating growing operator.

Tournament selection in EA is employed to choose a better parent structure by comparing the pair of individuals randomly picked from the population, which is used to generate the next generation population. The other genetic operators of crossover and mutation are used to further evolve the population. The crossover operator conducts an “inter-breeding” where a “branch” of two parent molecules are swapped and matched with the new moieties so that two new offspring molecules are generated (Figure 11). The mutation operator tends to execute a local modification or introduce a new moiety to replace the present part on a selected parent molecule to create a new molecule (Figure 12). It is worth noting that the growing operator can still be partly used within the next generations to maintain enough diversity of the population. The frequencies of using different operators can be set by the users in MoleGear.

### 3.3. Conformational Search

The Java package of Balloon uses distance geometry to generate an initial conformer, which is subjected to geometric modification by a genetic algorithm under the restraint of a MMFF94 force field [25]. Open Babel [26] (searches lowest-energy conformer based on the universal force field (UFF)) is often used to translate molecules between different formats. MolConverter (ChemAxon, Hungary) generates 3D coordinates from a Minkowski-like space followed by 3D geometry optimization to reach a local energy minimization using the Dreiding force field. A deeper conformational search using the same force field by the Cxcalc program (ChemAxon) can be further conducted. All these methods are integrated into MoleGear.

### 3.4. Fitness Function

New molecules generated by molecular assembly program are scored by fitness functions, and the more competitive candidates will stay inside the chemical space. By default, EA makes no assumptions about the fitness landscape; this generality makes it suitable for both receptor-based design and ligand-based design. Currently, MoleGear supports the receptor-based scoring by handling the output of the default docking programs AutoDock and Vina. Whereas AutoDock (from version 4.0.0) implements a Lamarckian genetic algorithm (LGA) search method that integrates a semiempirical free energy force field function for scoring of searched conformers, while Vina uses a gradient optimization method for conformer scoring by using an advanced knowledge-based and empirical function. Both software deal with the full ligand flexibility and the limited receptor (residue) flexibility and returns promising bound conformations together with predicted binding free energies.

The docking score (*Score_i,dock_*) for the *i^th^* molecule in MoleGear is defined as follows:

(1) $$Score_{i,dock}=\{\begin{array}{c} 1\;\;\;\;\;\;\;\;\;\;\;\;\;\;\;\;\;\;\;\;\;\;\;\;if\;E_{i}+PRESS_{e}\geq E_{max}\\ 1-\frac{E_{i}}{E_{min}-PRESS_{e}}\;\;\;if\;E_{min}<E_{i}+PRESS_{e}<E_{max}\\ 0\;\;\;\;\;\;\;\;\;\;\;\;\;\;\;\;\;\;\;\;\;\;\;if\;E_{i}+PRESS_{e}\leq E_{min} \end{array}$$

(2) $$PRESS_{e}=\left(E_{max}-E_{min}\right)\times c_{e}$$

where *E_i_* is the binding free energy of the *i^th^* molecule, *E_max_* and *E_min_* are the maximal and minimal energy of individuals in the current generation population, respectively, and *c_e_* is a user-defined strength coefficient. The score is scaled between 0 and 1. The lower the score, the better the fitness of a molecule. If the 3D structure of the biological receptor is unavailable, the ligand-based scoring is used as an alternative strategy based on the known active binding ligands. The ligand-based evaluation in MoleGear is implemented by analysis of similarity between template and novel structures expressed by their Euclidian distance in a proper space. The similarity score for the *i^th^* molecule (*Score_i,sim_*) is calculated by,

(3) $$Score_{i,sim}=\{\begin{array}{c} 1\;\;\;\;\;\;\;\;\;\;\;\;\;\;\;\;\;\;\;\;\;\;\;\;if\;D_{i}+PRESS_{d}\geq D_{max}\\ 1-\frac{D_{min}-PRESS_{d}}{D}\;\;\;if\;D_{min}<D_{i}+PRESS_{d}<D_{max}\\ 0\;\;\;\;\;\;\;\;\;\;\;\;\;\;\;\;\;\;\;\;\;\;\;if\;D_{i}+PRESS_{d}\leq D_{min} \end{array}$$

(4) $$PRESS_{d}=\left(D_{max}-D_{min}\right)\times c_{d}$$

where *D_i_* is the Euclidian distance between the *i^th^* molecule and the template molecule. *D_max_* and *D_min_* are, respectively, the maximal and minimal distance values of individuals in the current generation population, and *c_d_* is a user-defined strength coefficient. The score returns a number between 0 and 1, and the lower scores point to the more fit molecules. Moreover, MoleGear also supports the definition of a weighted-sum fitness function combing multiple objective terms:

(5) $$Score_{i}=\sum_{p}W_{p}\times Score_{i,p}$$

*Score_i_* and *w_p_* are the calculated fitness score and the weight applied to the *p^th^* property (e.g., docking and similarity) for the *i^th^* molecule, respectively. The weights are determined in accordance with the relative importance of the properties defined by users. Once a new generation population is built up by inserting new offspring molecules, the maximal and minimal property values (e.g., energy or distance) of the population will be retrieved, and the scores of individuals in the population will be updated according to the new intervals.

### 3.5. Fragment Library Design

Fragments derived from the existing drugs and compounds with known activities and properties make it likely to produce new compounds with feasible molecular structures. In addition, those fragments are also more likely to be “drug-like” molecules compared to random structures by satisfying the “rule of five” (Ro5) [43], and containing no reactive functional groups [13]. Thus, fragment-based drug design becomes a popular approach in drug design. However, the number of available drugs is vast, and often a small fraction of them is accessible. MoleGear offers a platform to build up a fragment library by the fragmentation of available molecular structures through splitting and screening operations.

*Splitting*: Molecular structures are hydrogen depleted and split into fragments at rotatable and non-terminal bonds (i.e., single bonds that are neither a part of a ring nor the ones that include atoms connected to only one other atom), or at variation sites of a common skeleton among a series of compounds of the same family. Prior to that, the rule of Ro5 is implemented to prevent the regeneration of undesirable structures. When MoleGear starts to split a structure, the bonds that connect to rings have a higher priority to be operated compared to those that connect to general atoms. The resulting fragments are saved in MDL as “sdf” format files with the atomic coordinates and substitution points (R-groups) indicated.

**Figure 13:**
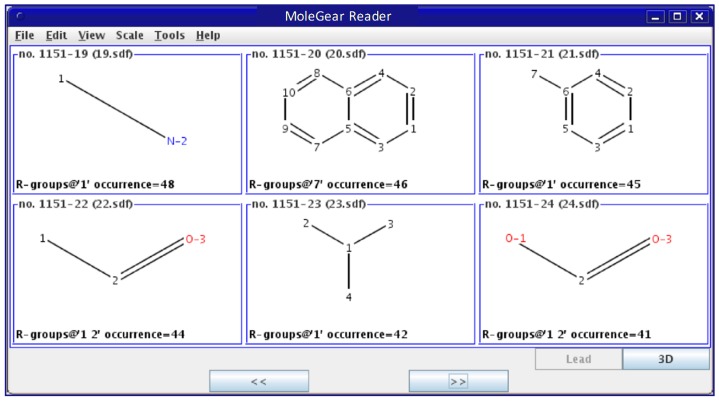
Graphical view of a fragment library built by MoleGear.

*Screening*: All derived fragments are subjected to certain filter rules, so duplicate and unfavorable moieties will be removed. The so-called “unfavored” structures are dependent on the specific definition by the users. A library is then established, which contains preferable building blocks as scaffolds and side chains. A link file containing the paths to the fragments and their occurrences in known molecules is then created. It is useful to present all the structures in a united graphical interface (Figure 13) so that the user can more easily check and refine the dataset.

### 3.6. Chemical Space Analysis

A set of molecular descriptors (e.g., electronic, geometrical, topological, and hybrid categories) imported from the CDK QSAR package are used to capture the chemical features of an involved molecule or fragment set. This usually results in a multi-dimensional data space spanned with many correlated variables. MoleGear uses principal component analysis (PCA) to convert the multi-dimensional descriptor space into a lower-dimensional space spanned with independent principle properties. The objects in the principle property space can be visualized through score plotting. Various selection algorithms (e.g., most descriptive compound (MDC) [44] and D-optimal design) can be used to select a subset of molecules that spans an important structural or physiochemical space of the original dataset. The selected structures can be plotted with the unselected ones distinguishingly in the score space. Regression by means of projections to latent structures (PLS) method, and classification by k-nearest neighbors (K-NN) can be further investigated by QSAR/QSPR analytical tools.

## 4. Conclusions

The Java-based platform MoleGear was developed for de novo molecular design by a well-schemed evolutionary algorithm, which uses a graph-based method to represent molecular structures. A suite of fragment-based operators is used to assemble novel molecules, and various strategies are applied to score the assembled structures. The EA in MoleGear can be implemented in parallel over multiple nodes, and thus enables large-scale optimizations. In addition, the complementary utilities such as fragment library design, chemical space analysis, and graphical view are well supported. The functionality and flexibility of MoleGear has been demonstrated by different designs of drug-like inhibitors for HIV-1 protease. The designed candidate molecules were found to be similar with the reference molecule in both structure and conformation, which indicates that MoleGear can be used for de novo design of drug-like molecules. Moreover, it is also expected that the designed molecules are chemically feasible. The evolutionary de novo molecular design is implemented on Java-based platform, and can be further expanded to integrate extra specific functionalities.

## Figures and Tables

**Table 1:** Molecular descriptors used in ligand-based design of the novel HIV-1 protease inhibitors.

Category	Molecular Descriptors
3D	Charged partial surface area (CPSA) [34]
	Gravitational index [35]
	Molecular length to breadth ratio
	Molecular distance edge (MDE) [36]
	Moment of inertia
	Geometrical shape coefficients of radius–diameter diagram [37]
	Weighted holistic invariant molecular (WHIM) descriptors [38]
2D	Topological polar surface area (TPSA) [39]
	Topological shape coefficients of radius–diameter diagram
	XLogP [40]
	Polarizability differences between all bonded atoms
	Numbers of hydrogen bond acceptors
	Numbers of hydrogen bond donor
	Numbers of atoms
	Numbers of bonds
